# Comparative karyotype analysis and chromosome evolution in the genus *Aplastodiscus* (Cophomantini, Hylinae, Hylidae)

**DOI:** 10.1186/1471-2156-13-28

**Published:** 2012-04-20

**Authors:** Simone Lilian Gruber, Juliana Zina, Hideki Narimatsu, Célio Fernando Baptista Haddad, Sanae Kasahara

**Affiliations:** 1 UNESP, Universidade Estadual Paulista, Instituto de Biociências, Departamento de Biologia, Av. 24A, 1515, 13506-900, Rio Claro, SP, Brazil; 2 Universidade Estadual do Sudoeste da Bahia, Departamento de Ciências Biológicas, Rua José Moreira Sobrinho, s/n, 45206-000, Jequié, BA, Brazil; 3 UNESP, Universidade Estadual Paulista, Instituto de Biociências, Departamento de Zoologia, Av. 24A, 1515, 13506-900, Rio Claro, SP, Brazil

**Keywords:** Amphibian, BrdU, FISH, Ag-NOR, C-band, CMA_3_, Phylogeny

## Abstract

**Background:**

The frogs of the Tribe Cophomantini present, in general, 2n = 24 karyotype, but data on *Aplastodiscus* showed variation in diploid number from 2n = 24 to 2n = 18. Five species were karyotyped, one of them for the first time, using conventional and molecular cytogenetic techniques, with the aim to perform a comprehensive comparative analysis towards the understanding of chromosome evolution in light of the phylogeny.

**Results:**

*Aplastodiscus perviridis* showed 2n = 24, *A. arildae* and *A*. *eugenioi*, 2n = 22, *A*. *callipygius*, 2n = 20, and *A*. *leucopygius*, 2n = 18. In the metaphase I cells of two species only bivalents occurred, whereas in *A. arildae*, *A*. *callipygius*, and *A*. *leucopygius* one tetravalent was also observed besides the bivalents. BrdU incorporation produced replication bands especially in the largest chromosomes, and a relatively good banding correspondence was noticed among some of them. Silver impregnation and FISH with an rDNA probe identified a single NOR pair: the 11 in *A*. *perviridis* and *A. arildae*; the 6 in *A. eugenioi*; and the 9 in *A. callipygius* and *A. leucopygius*. C-banding showed a predominantly centromeric distribution of the heterochromatin, and in one of the species distinct molecular composition was revealed by CMA_3_. The telomeric probe hybridised all chromosome ends and additionally disclosed the presence of telomere-like sequences in centromeric regions of three species.

**Conclusions:**

Based on the hypothesis of 2n = 24 ancestral karyotype for *Aplastodiscus*, and considering the karyotype differences and similarities, two evolutionary pathways through fusion events were suggested. One of them corresponded to the reduction of 2n = 24 to 22, and the other, the reduction of 2n = 24 to 20, and subsequently to 18. Regarding the NOR, two conditions were recognised: plesiomorphy, represented by the homeologous small-sized NOR-bearing pairs, and derivation, represented by the NOR in a medium-sized pair. In spite of the apparent uniformity of C-banding patterns, heterogeneity in the molecular composition of some repetitive regions was revealed by CMA_3_ staining and by interstitial telomeric labelling. The meiotic tetravalent might be due to minute reciprocal translocations or to non-chiasmatic ectopic pairing between terminal repetitive sequences. The comparative cytogenetic analysis allowed to outline the chromosome evolution and contributed to enlighten the relationships within the genus *Aplastodiscus*.

## Background

The original description of *Aplastodiscus* Lutz, 1950, in the family Hylidae, was based on the species *A*. *perviridis*. However, many questions regarding the taxonomy of this genus remained, because the traits used for its characterisation were shared with representatives of the genus *Hyla*[1]. This fact led to the assignment of the name *Hyla perviridis*[2], and this species was included in the *H. albomarginata* group, along with *H. albomarginataH*. *albosignata*, and *H*. *albofrenata*, due to, among other characters, the green colour typical of the species [3].

Based on morphological and bioacoustics data, as well as breeding behaviour of *Hyla cochranae* and *H*. *perviridis*[1], the genus *Aplastodiscus* was re-characterised, but the authors emphasised that further taxonomic studies were still necessary. Later, based on reproductive mode, it was suggested that the *Hyla albosignata* and *H*. *albofrenata* species complexes should be included in the genus *Aplastodiscus*[4]. Subsequently, comprehensive reviews of the taxonomy and phylogeny of the family Hylidae were performed [5,6], confirming the previous suggestion [4]. The 15 known species of *Aplastodiscus* are currently distributed into three groups: the *A*. *albofrenatus* group (*A*. *albofrenatusA*. *arildaeA*. *ehrhardtiA*. *eugenioiA*. *weygoldti*, and *A*. *musicus*), the *A*. *albosignatus* group (*A*. *albosignatusA*. *callipygiusA*. *cavicolaA*. *flumineusA*. *ibirapitangaA*. *leucopygius*, and *A*. *sibilatus*), and the *A*. *perviridis* group (*A*. *cochranae* and *A*. *perviridis*) [7].

About half of the known species of *Aplastodiscus* have been karyotyped and the former analysis, based only on standard staining, showed 2n = 24 and 2n = 22 in *A*. *albofrenatus*, and 2n = 20 and 2n = 18 in *A*. *albosignatus*, collected in distinct Brazilian localities [8]. The author admitted that the different karyotypes might correspond, in fact, to distinct species.

Recently, four species of *Aplastodiscus* with 2n = 22, i.e., *A*. *albofrenatusA*. *arildaeA*. *ehrhardti,* and *A*. *eugenioi* were karyotyped and some species-specific chromosome markers were found [9]. Analysing specimens of *A*. *perviridis* and *A*. *cochranae* with 2n = 24, *A*. *albosignatus* with 2n = 20, and *A*. *leucopygius* with 2n = 18, the same authors suggested that the karyotype differentiation of these species might have resulted from a reduction in the number of the small-sized chromosomes [10]. These data confirmed the karyotype variability in *Aplastodiscus*, an unusual finding in anurans, which are characterized, in general, by conserved chromosome constitution [11,12].

In the present paper, a comprehensive comparative analysis was carried out for the first time based on five species of *Aplastodiscus* with distinct diploid numbers, one of them (*A*. *callipygius*) never karyotyped before. Besides Ag-NOR impregnation, C-banding, and FISH with probes of rDNA and of telomeric repeats, which had been previously used for some species [9,10], the chromosomes of our sampled species were also analysed with fluorochrome staining and replication-banding after BrdU incorporation. The aim was to search for additional markers, towards a better understanding of chromosome evolution in light of the phylogeny [5,6], contributing to make clear the relationships within the genus *Aplastodiscus*.

## Methods

### Analysed species

Cytogenetic analyses were performed on 28 individuals representing five species of *Aplastodiscus* (Table 1), collected in the states of São Paulo (SP) and Minas Gerais (MG). The animals were identified and deposited in the amphibian collection Célio Fernando Baptista Haddad (CFBH) housed in the Department of Zoology, UNESP, Rio Claro, SP, Brazil.

**Table 1:** Species, number of individuals, sex, voucher number, and collecting localities in Brazil

**Species**	**Number**	**Sex**	**Voucher number (CFBH)**	**Collecting localities**
*Aplastodiscus perviridis*	**4**	males	22394, 22395, 22401, 22402	Camanducaia, MG
*Aplastodiscus arildae*	**1**	male	22387	Serra do Japí, Jundiaí, SP
	**4**	males	28582, 30409, 30410, 30411	Mogi das Cruzes, SP
*Aplastodiscus eugenioi*	**2**	male, female	22373, A505	Ubatuba, SP
*Aplastodiscus callipygius*	**7**	males	7514, 7515, 7516, 22396, 22397, 22403, 22404	Camanducaia, MG
*Aplastodiscus leucopygius*	**3**	males	22389, A732, A733	Serra do Japí, Jundiaí, SP
	**1**	female	22388	
	**6**	males	28583, 28584, 30412, 30413, 30414, 30415	Mogi das Cruzes, SP

CFBH: Célio Fernando Baptista Haddad Collection, UNESP, Rio Claro, SP, Brazil.

### Chromosome preparation and cytogenetic technique

Direct cytological preparations were obtained from bone marrow, liver, and testes [13] and from intestinal epithelium [14]. In vivo treatment with 5-bromodeoxiuridine (BrdU) was carried out for some specimens [15]. The slides were standard stained with Giemsa, and submitted to Ag-NOR technique [16], C-banding [17], fluorochrome staining with AT-specific DAPI and GC-specific CMA_3_[18], and replication band differentiation using Fluorochrome Plus Giemsa (FPG) or Acridine Orange [19,20]. The ribosomal probe HM123 [21] was hybridised using the fluorescence in situ hybridisation (FISH) technique [22] and a telomeric probe, following the manufacturer's manual (Dako Cytomation Denmark A/S Kit). The bi-armed chromosomes were classified as metacentric, submetacentric, or subtelocentric by visual inspection, following the nomenclature of Green and Sessions [23,24].

## Results

### Karyotype constitution and meiosis

**Figure 1:**
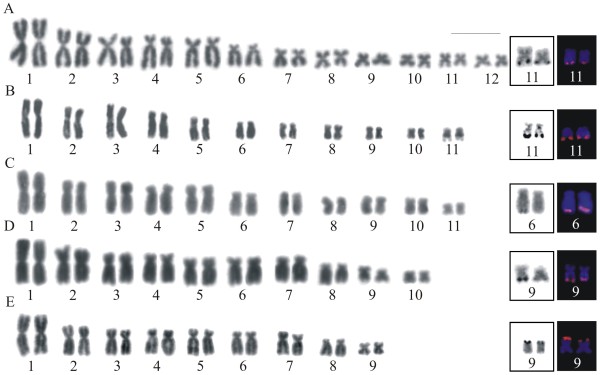
**Giemsa-stained karyotypes of* Aplastodiscus. * A. *** A. perviridis *, male, 2n = 24; **B.***A. arildae*, male, 2n = 22; **C.***A. eugenioi*, male, 2n = 22; **D.***A. callipygius*, male, 2n = 20. **E.***A. leucopygius*, male, 2n = 18. Insets show marker pairs, visualised by Ag-NOR and FISH with the rDNA probe. Bar = 10 μm.

The specimens of *A*. *perviridis* showed 2n = 24, FN = 48 (Figure 1A), and a karyotype formed by five large pairs with slight variation in size from pairs 2 to 5, one medium pair 6, and six small pairs 7 to 12, with subtle variation in size. Pair 1 was metacentric, pairs 2, 3, 4, and 5 were submetacentric, pair 6 was subtelocentric, and the remaining pairs were classified as metacentric or submetacentric. *Aplastodiscus arildae* and *A*. *eugenioi*, with 2n = 22, FN = 44 (Figure 1B, 1C), had very similar karyotype constitution compared with *A*. *perviridis*, except that the small-sized group included five pairs 7 to 11 and that pair 2 was clearly metacentric. *Aplastodiscus callipygius* and *A*. *leucopygius*, with 2n = 20, FN = 40, and 2n = 18, FN = 36, respectively (Figure 1D, 1E), exhibited large-sized pairs 1 to 7, with slight variation from 2 to 7, one medium pair 8, and two small-sized pairs 9 and 10 in *A. callipygius*, and only one small-sized pair 9 in *A*. *leucopygius*. Pair 1 was metacentric, pairs 2 to 7 were submetacentric, pair 8 was subtelocentric, and the remaining pairs were metacentric or submetacentric in both species.

Secondary constriction was noticed in one or both homologues of chromosome pair 11 in *A*. *perviridis* and *A*. *arildae*, as well as in one or both homologues of chromosome pair 9 in *A*. *callipygius* and *A*. *leucopygius*. No sex-related chromosome heteromorphism was observed in male (XY) or female (ZW) of *A*. *eugenioi* and *A*. *leucopygius*; neither in males (XY) of the remaining species.

**Figure 2:**
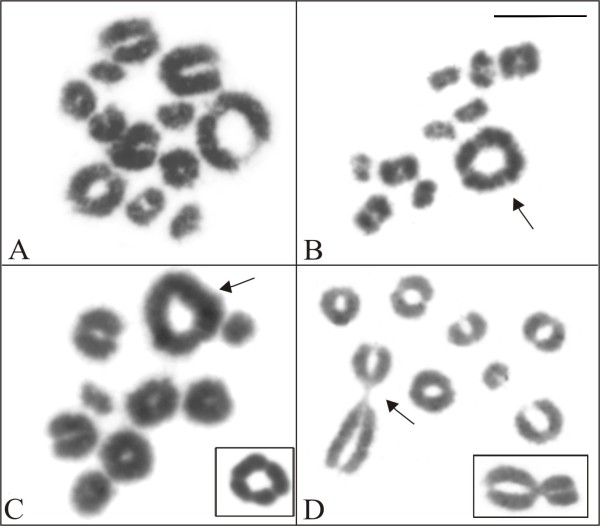
**Giemsa-stained meiotic cells of* Aplastodiscus. *A.** metaphase I of *A. perviridis*, with 12 bivalents; **B.** metaphase I of *A. arildae*, with nine bivalents and one quadrivalent (arrow); **C.** metaphase I of *A. callipygius*, with eight bivalents and one quadrivalent (arrow and inset); **D.** metaphase I of *A. leucopygius*, with nine bivalents (arrow and inset, connected bivalents). Bar = 10 μm.

In metaphase I cells of *A*. *perviridis* (Figure 2A) and *A*. *eugenioi*, 12 and 11 bivalents, respectively, were observed, while during metaphase II, 12 chromosomes were observed in the former species; for *A*. *eugenioi* this meiotic stage was not available. In *A*. *arildae* and *A. callipygius*, diplotene and metaphase I cells invariably showed one tetravalent, plus 9 and 8 bivalents, respectively (Figure 2B, 2C). *Aplastodiscus callipygius* exhibited 10 chromosomes in metaphase II cells, but this meiotic stage was not available for *A. arildae*. In metaphase I cells of *A. leucopygius* two of the nine bivalents appeared to be connected (Figure 2D), and during the metaphase II, 9 chromosomes were observed.

### Differential staining and FISH

The technique of nucleolar organiser region by silver impregnation was performed in almost all individuals of the sampled species, excepting in two individuals of *A*. *callipygius*, showing a single pair of Ag-NOR: at the terminal region of the long arms of chromosome 11 in *A*. *perviridis* and *A*. *arildae*, at the terminal region of the long arms of chromosome 6 and 9 in *A*. *eugenoi* and *A*. *callipygius*, respectively, and at the terminal region of the short arms of chromosome 9 in *A*. *leucopygius* (Figure 1). One single Ag-NOR, as shown in Figure 1C for *A*. *eugenioi*, was observed eventually in metaphases of some of the individuals in all analysed species. The sites of Ag-impregnation were coincident with the secondary constrictions in most cases. The FISH technique carried out in one single individual of each species confirmed that ribosomal sequences were in the sites previously identified by silver impregnation, always in the two homologues of the corresponding NOR-bearing pair (Figure 1).

**Figure 3:**
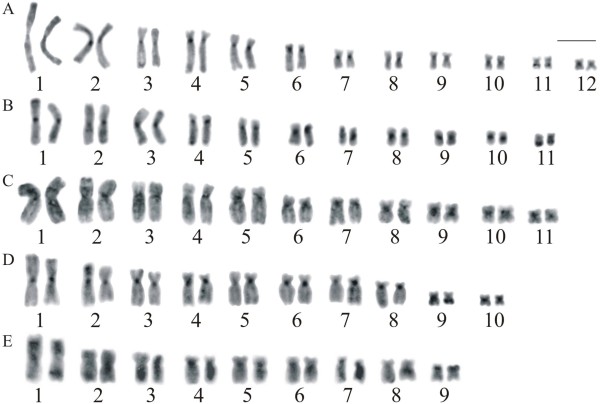
**C-banded karyotypes of* Aplastodiscus. *A.*** A. perviridis *; **B.*** A. arildae *; **C.*** A. eugenioi *; **D.*** A. callipygius *; **E.*** A. leucopygius *. Bar = 10 μm.

**Figure 4:**
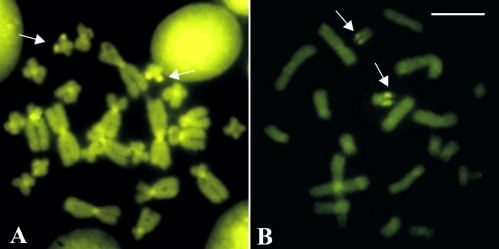
**CMA**_**3**_**-stained metaphases of* Aplastodiscus *.A.*** A. perviridis *; **B.*** A. arildae *. Bright CMA_3_ fluorescence at the NOR site (arrow) and in A, also in the centromeric region of the chromosomes. Bar = 10 μm.

The heterochromatin in all species had a predominantly centromeric distribution, with additional labelling at the NOR site (Figure 3), but in some metaphases this C-band was very slight or not well visualised as in the Figure 3C for *A*. *eugenioi*. Fluorochrome staining was carried out in four species, with exception of *A. eugenioi*. In *A. perviridis*, bright fluorescence was observed with CMA_3_ in the NOR and in the centromere of all chromosomes, but less brilliant in the small-sized chromosomes (Figure 4A). Fluorescence at the NOR site was also observed in *A*. *arildae* (Figure 4B), *A. callipygius*, and *A. leucopygius* (data not shown). When stained with DAPI, no particular brilliant region was observed in the chromosomes of any of the four species. 

**Figure 5:**
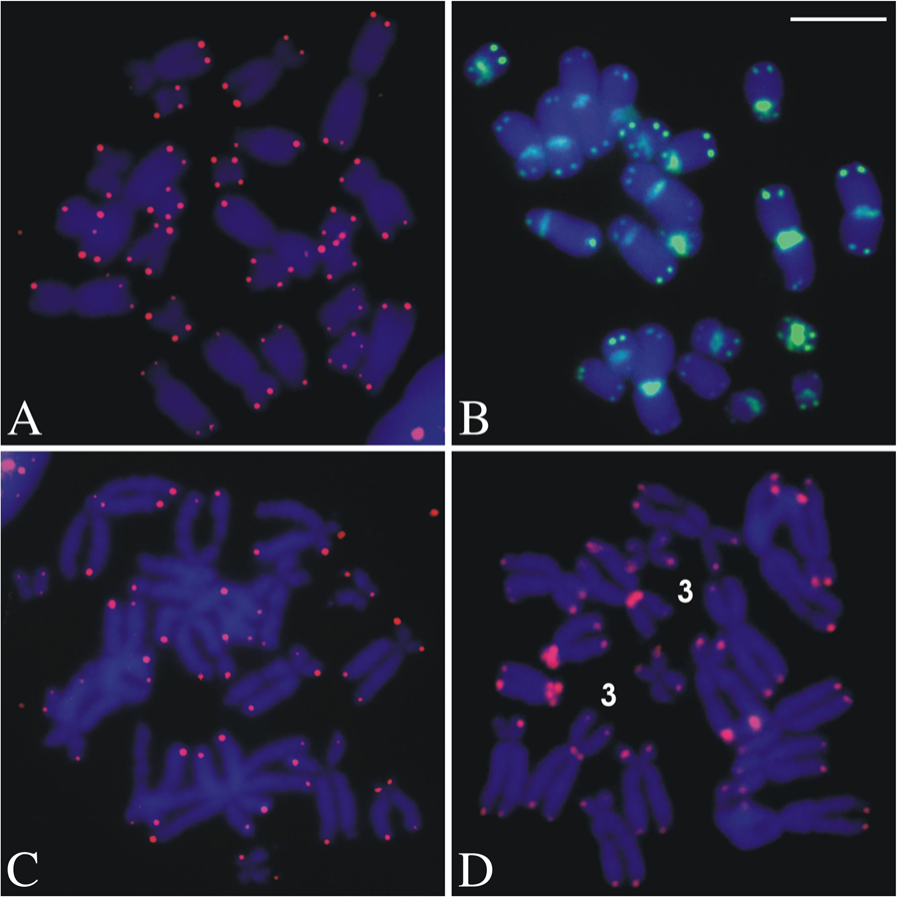
**FISH using a telomeric probe in metaphases of* Aplastodiscus. *A.*** A. perviridis *; **B.*** A. arildae *; **C.*** A. callipygius *; **D.*** A. leucopygius *. A hybridisation signal is seen in the centromeric region of chromosome 3 of *A. leucopygius* and in the chromosomes of *A. arildae*. Bar = 10 μm.

Telomeric probe hybridised all chromosome ends in *A*. *perviridis* and *A*. *callipygius* (Figure 5A, 5CS), whereas in *A. leucopygius* additional labelling of interstitial telomeric sequence (ITS) was observed at the centromere region of chromosome 3 (Figure 5D). In *A*. *arildae* (Figure 5B) and *A. eugenioi*, hybridisation occurred in the telomeres and centromeres of all chromosomes, although the fluorescent labelling was subtle in the latter species.

**Figure 6:**
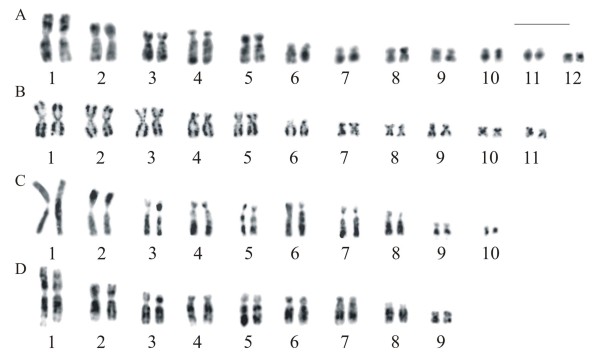
**Replication-banded karyotypes of* Aplastodiscus *, after BrdU incorporation. A.*** A. perviridis *; **B.*** A. arildae *; **C.*** A. callipygius *; **D.*** A. leucopygius *. Bar = 10 μm.

Incorporation by BrdU carried out in *A*. *perviridis*, *A*. *arildae*, *A*. *callipygius*, and *A. leucopygius* produced replication bands especially in the largest chromosomes, whereas the smallest chromosomes showed poor differentiation (Figure 6). In spite of the distinct degrees of banding differentiation, tentative inter-specific comparisons were performed among the large and medium-sized chromosomes, showing relatively good banding correspondence among some chromosomes (e.g., the 1 and the 5 of the four species, the 2 and the 3 of *A*. *perviridis*, *A*. *callipygius*, and *A. leucopygius*, the 6 of *A*. *perviridis* and the 8 of *A. leucopygius*), and roughly the same banding feature for some others (the 4 of the four species).

## Discussion and Conclusions

The present cytogenetic analysis confirmed the variability of 2n = 24, 2n = 22, 2n = 20, and 2n = 18 within the genus *Aplastodiscus*, contrary to that is commonly observed in the subfamily Hylinae, in general with invariable 2n = 24 karyotypes [25]. Distinct diploid numbers in *Aplastodiscus* were originally reported [8] and, more recently, the sample of karyotyped species was enlarged [9,10]. Taking into account our report on *A. callipygius*, analysed here for the first time, a total of nine representatives of the genus now have described karyotypes: *A. cochranae* and *A. perviridis* (*A. perviridis* group) with 2n = 24; *A. albofrenatusA. arildaeA*. *ehrhardti*, and *A. eugenioi* (*A. albofrenatus* group) with 2n = 22; *A. albosignatus* and *A. callipygius* with 2n = 20; and *A. leucopygius* with 2n = 18 (*A. albosignatus* group).

It is important to emphasise that several individuals of these species have been collected in more than one locality and no karyotype intraspecific variation in the chromosome number has been found except, at the first sight, in the sample of *A*. *albofrenatus* (2n = 24 in Floresta da Tijuca, Rio de Janeiro, RJ, and 2n = 22 in Boraceia, SP) and *A. albosignatus* (2n = 20 in Boraceia, SP, and 2n = 18 in Teresópolis, RJ) [8], although this variation was probably consequence of misidentification, according to the author. Later, Carvalho et al. [9,10], based on the geographical distribution of *Aplastodiscus* and on the cytogenetic data of some individuals collected in the same or near the localities screened by Bogart [8], concluded that the formerly karyotyped specimens were actually misidentification, and suggested that the animals with 2n = 22, 2n = 20, and 2n = 18 corresponded to *A*. *arildaeA*. *albosignatus*, and *A*. *leucopygius*, respectively. Additionally, considering that the range of *A. perviridis* includes the state of Rio de Janeiro [7] and that the diploid number for this species is 2n = 24 [10, present work], the *A. albofrenatus* from Tijuca Forest, RJ [8], could be *A. perviridis* or some other species of *Aplastodiscus*.

Observing the karyotypes analysed so far with standard Giemsa staining, it was noticed that, although the ordering or the nomenclature adopted for each chromosome pair could differed among the authors, the chromosome constitution was equivalent within each group of species. Even presenting distinct diploid numbers, some shared characteristics could be recognized: the first five chromosome pairs in *A*. *perviridis*, *A. callipygius*, and *A. leucopygius* were equivalent in morphology and relative size; similarity also exists for the first five pairs of *A. arildae* and *A. eugenioi*, both exhibiting 2n = 22 karyotypes, but their pair 2 is clearly metacentric and larger than the 2 observed in the three former species; all six species exhibited a subtelocentric medium-sized marker but in distinct positions in the karyograms, that is, in the majority of the species the marker was the pair 6, whereas in *A. callipygius* and *A. leucopygius* it corresponded to pair 8. On the other hand, some conspicuous karyotype differences could be pointed out: a progressive reduction in the number of the small pairs, totalling six in *A. perviridis*, five in *A. arildae* and *A. eugenioi*, two in *A. callipygius*, and one in *A. leucopygius*; the presence in *A. callipygius* and *A. leucopygius* of two large-sized chromosome pairs 6 and 7, not observed in any other karyotype.

**Figure 7:**
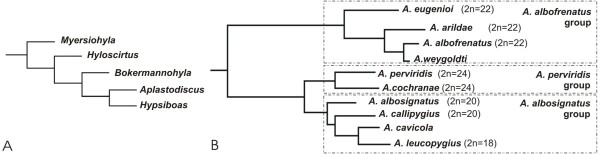
**Adapted phylogenies.A.** phylogenetic tree of Tribe Cophomantini (Hylinae, Hylidae) based on Faivovich et al. [5], showing the position of *Aplastodiscus* and related genera *Hypsiboas* and *Bokermannohyla*; **B.** phylogenetic tree of genus *Aplastodiscus* based on Wiens et al. [6], with indication of the known diploid number of each karyotyped species [9, 10, present work].

Taking into account that 2n = 24 was considered a synapomorphy for Hylinae [5], most probably the ancestor of *Aplastodiscus* had 24 chromosomes (Figure 7A), and the karyotype constitution would be equivalent to that observed in the related genera *Bokermannohyla* and *Hypsiboas*, as well as to that of the 2n = 24 *A*. *cochranae* and *A. perviridis*. Therefore, the chromosome evolution within the genus *Aplastodiscus* occurred primarily by reducing the diploid number from an ancestor with 2n = 24 due to chromosome fusions. However, replication banding data obtained for the first time in species of *Aplastodiscus* could not be used for identifying the probable structural rearrangements, although correspondence of banding patterns had been confirmed among some chromosomes. 

Other analysis provided additional data on the karyotype variability within the genus *Aplastodiscus*. Both Ag-impregnation and FISH with an rDNA probe confirmed one pair of NOR-bearing chromosomes for all species. The eventual heteromorphism of Ag-NOR, that is, presence and lack of labelling in metaphases of some of the individuals in all analysed species, was interpreted as result rather from a differential activity than from the deletion in the amount of rDNA repeats, since two FISH signals, equivalent in size in both homologues, were observed in all cases. So, the transcriptional activity of rDNA might be inactivated or to be too low to be detected by silver impregnation in some chromosomes.

In *A. perviridisA. arildaeA. callipygius*, and *A. leucopygius*, the NOR was located in a homeologous small-sized chromosome, although corresponding to the 11 in the two former species and to 9 in the two latter, due to the reduction in the diploid number. This condition of NOR in one of the smallest chromosome pairs was also observed in other species of *Aplastodiscus*[9,10], as well as in the hylids of genera *BokermannohylaHylaHypsiboas*, and those belonging to *Scinax* of *rubber* clade [25-32], and this can be considered a plesiomorphy for the family. These marker chromosome pairs are most probably homeologous, although with non-coincident position in the case of the karyograms of species with the same chromosome number.

In *A. eugenioi* of the present sample and from the literature [9], the NOR had a derived location, in a medium-pair 6, or pair 7 in the case of *A. ehrhardti* and *A*. *albofrenatus*, but the latter species had an additional NOR site in chromosome 1 [9]. In the three species the medium-sized pairs bearing NOR, referred by us as the 6, were probably the same, and this condition may constitute a synapomorphy. Gross structural rearrangement seemed not to be the mechanism underlying the change of NOR from a small-sized to a medium-sized chromosome, because the chromosome 6 was always recognized as a subtelocentric marker in all species, independently if bearing or not NOR. Minute structural rearrangements, transposition by means of mobile elements or other mechanisms were not discarded, but they were not demonstrated through the used banding techniques. These mechanisms would also explain the change of NOR from the long to the short arms of chromosome 9 in *A. leucopygius*.

All the sampled species of *Aplastodiscus* had similar heterochromatin distribution, with C-bands in the centromeres and at the NOR sites. Our data on *A. perviridisA*. *arildae*, and *A*. *eugenioi* differed from the C-banding pattern of the corresponding species previously analysed [9,10], that demonstrated additional secondary C-positive regions in some chromosome pairs. This might be indicative of population difference or even be result of technical procedures. In spite of the apparent uniformity in the C-banding, an unequivocal molecular heterogeneity of the heterochromatin was revealed by CMA_3_ staining and FISH with a telomeric probe. In fact, the centromeric heterochromatin of the chromosomes of *A. perviridis* was GC-rich repetitive region, as shown by its bright fluorescence after CMA_3_ staining. On the other hand, the hybridisation of the telomeric probe outside of the ends of all chromosomes in *A. arildae* and *A. eugenioi*, and chromosome 3 in *A. leucopygius*, indicated the presence of repeats similar to (TTAGGG)_n_ in the centromeric region. Another possible type of centromere repetitive region corresponded to that of the chromosomes of *A. callipygius* and *A. leucopygius*, since neither the base-specific fluorochromes nor the telomeric probe yielded a fluorescent labelling.

Occasionally, interstitial hybridisation of the telomeric probe may represent true vestiges of telomeres, corroborating structural rearrangements occurred during chromosome evolution, as described in rodents [33,34]. Nevertheless, this possibility was excluded in the *Aplastodiscus* species [9, present work], and in other frogs presenting ITS [35,36]. Regardless, the presence of repetitive DNA bearing telomere-like sequences outside the telomeres might represent an additional cytological marker for species or even species groups.

The meiotic analysis in *A. arildaeA. callipygius*, and *A. leucopygius* confirmed the occurrence of multivalent chromosome pairing, as described in *A. albofrenatus* and *A. arildae*[9]. While in our sample of *A. arildae* and *A. callipygius* a clear tetravalent pairing was seen, in *A. leucopygius* the tetravalent figure was not characteristic, because the involved chromosomes formed two recognizable bivalents. In all these three species, the chromosomes of the largest pair were involved in the tetravalent.

In vertebrates, including frogs [37-40], rings or chains of meiotic multivalents have been reported. The most illustrative case among animals was described in *Ornithorhynchus anatinus*[41], in which the multivalent formation was attributed to sequential reciprocal translocations. The same occurred in one specimen of the frogs *Haddadus binotatus*[39] and *Leptodactylus pentadactylus*[40], which presented meiotic chain and several odd heteromorphic chromosomes in their karyotypes.

In our study there was no evidence of reciprocal translocation to explain the tetravalent formation, unless it involved minute segments, not detected by the used banding techniques. Another explanation would be the non-chiasmatic ectopic pairing between terminal repetitive sequences of non-homologous chromosomes, proposed by Schmid et al. [12] as a reasonable alternative for similar cases described in the literature [9,37,38]. Our data gave no support to any of these hypothesis.

Our cytogenetic analysis on *Aplastodiscus* and the comprehensive comparative analysis allowed us to consider the following possible homeologies: chromosomes 1, 4, and 5 of *A*. *perviridis*, *A. arildae*, *A. callipygius*, and *A. leucopygius*; the chromosome 2 of *A*. *perviridis*, *A. callipygius*, and *A. leucopygius* with the chromosome 3 of *A. arildae* and *A. eugenioi*; the chromosomes 3 of *A*. *perviridis*, *A. callipygius*, and *A. leucopygius*; the chromosomes 6 and 11 of *A*. *perviridis*, *A. arildae*, and *A. eugenioi* with the chromosomes 8 and 9, respectively, of *A. callipygius* and *A. leucopygius*; the chromosomes 7, 8, 9, and 10 of *A*. *perviridis*, *A. arildae*, and *A. eugenioi*; and the chromosome 12 of *A*. *perviridis* with the chromosome 10 of *A. callipygius*. The corresponding chromosome 2 of *A. arildae* and *A. eugenioi*, and the chromosomes 6 and 7 of *A. callipygius* and *A. leucopygius* were interpreted as resulted of rearrangement. Based on these presumed data, the chromosome evolution in the genus *Aplastodiscus* from an ancestor with 2n = 24 was outlined. Nevertheless, two evolutionary pathways were proposed: one involving two fusions events, in which participate the small elements 7, 8, 9, and 10, giving rise to two new large-sized pairs 6 and 7, as in the karyotype with 2n = 20 of *A. callipygius* and with 2n = 18 of *A. leucopygius*; and the other, fusion involving the small chromosome 12 and the large chromosome 3, giving rise to the metacentric pair 2, as in the karyotypes with 2n = 22 of *A. arildae* and *A. eugenioi*. This hypothesis is supported by our present cytogenetic data, but undoubtedly, other resolute approaches (e.g., chromosome painting, gene linkage, among others) are still necessary in order to confirm the chromosome evolution within the genus *Aplastodiscus*.

Another achievement of the present study was the confirmation, by means of chromosome analysis, of the relationships among species or species groups of *Aplastodiscus*, as shown in the adapted phylogenetic tree based in Wiens et al. [6], and shown in Figure 7B. Including the known diploid numbers of all karyotyped species, the two pathways in the chromosome evolution were well visualised, and the cytogenetic data gave support to the molecular phylogeny and distribution of the species in the known groupings. Certainly, further species sampling, especially of those that have never been karyotyped, will be of great interest to confirm or not the relationships within the genus *Aplastodiscus*.

## Abbreviations

2n, diploid number; Ag-NOR, nucleolar organiser region marked by silver impregnation; BrdU, 5-bromodeoxiuridine; CMA_3_, Chromomycin A3; DAPI, 4′-6-diamidino-2-phenylindole; FISH, Fluorescent in situ hybridization; FPG, Fluorochrome Plus Giemsa; FN, Fundamental number; ITS, Interstitial telomeric sequence; NOR, Nucleolar organiser region; rDNA, Ribosomal DNA.

## Competing interests

Non-financial competing interests.

## Authors' contributions

SLG performed the cytogenetic studies and drafted the manuscript. JZ collected some animals and helped in the review of the manuscript. HN collected some animals and helped with identification. CFBH provided support on zoological information, carried out the species identification, and revised the manuscript. SK supervised the cytogenetic studies, participated in the draft, and in the revision of the final text. All authors read and approved the final manuscript.
